# Development and validation of a nomogram for predicting postoperative lower extremity deep vein thrombosis in patients with traumatic spinal fractures: a retrospective study

**DOI:** 10.7717/peerj.21184

**Published:** 2026-04-22

**Authors:** Guangxu Fu, Yong Wang, Dengwu Tan, Zhen Zhang, Xiaoyan Yu

**Affiliations:** 1 Department of Orthopedic Surgery, The People’s Hospital of Lichuan City, Enshi Tujia and Miao Autonomous Prefecture, China; 2 Department of Anesthesiology, The People’s Hospital of Lichuan City, Enshi Tujia and Miao Autonomous Prefecture, China

**Keywords:** Spinal fractures, Deep vein thrombosis, Risk prediction model, Postoperative complications, Nomogram

## Abstract

**Background:**

Patients undergoing surgery for traumatic spinal fractures are at high risk for postoperative venous thromboembolism (VTE), with deep vein thrombosis (DVT) incidence approaching 20% despite standard prophylaxis. This highlights the need for enhanced and specialized risk stratification. We aimed to develop and internally validate a preoperative prediction model for postoperative lower extremity DVT in this population.

**Methods:**

This retrospective cohort study analyzed data from 1,676 patients who underwent surgery for traumatic spinal fractures at a single center between January 2020 and March 2025. Postoperative DVT was diagnosed *via* ultrasonography. Predictors were identified from 29 candidate variables through univariate analysis, followed by stepwise multivariable logistic regression. Model performance was evaluated using discrimination (area under the receiver operating characteristic curve, AUC), calibration (calibration plot, Hosmer-Lemeshow test), and clinical utility (decision curve analysis, DCA). A nomogram was constructed for visualization.

**Results:**

The postoperative DVT incidence was 14.3% (239/1,676). Six independent preoperative predictors were identified: prolonged bed rest > 72 hours (adjusted odds ratio (aOR) = 5.208, 95% CI [3.319–8.171]), pre-existing lower extremity vascular disease (aOR = 2.938, 95% CI [1.641–5.258]), elevated D-dimer (aOR = 1.582 per mg/L, 95% CI [1.448–1.729]), elevated fibrinogen (aOR = 1.434 per g/L, 95% CI [1.138–1.807]), severe neurological impairment (American Spinal Injury Association (ASIA) Impairment Scale grade A/B), and advanced age (aOR = 1.019 per year, 95% CI [1.003–1.035]). The model exhibited robust discrimination (AUC: 0.891 in the training set, 0.885 in the testing set), excellent calibration (Hosmer-Lemeshow *P* > 0.7), high sensitivity (90.5–91.2%), and moderate specificity (74.3–74.5%). DCA confirmed its clinical utility across a wide range of threshold probabilities.

**Conclusion:**

This study developed and validated a novel preoperative nomogram for predicting postoperative DVT in patients undergoing surgery for traumatic spinal fractures. Incorporating six readily accessible variables, this tool enables individualized risk stratification and may inform targeted prophylactic strategies.

## Introduction

Traumatic spinal fractures represent a substantial global health burden, often leading to permanent disability and requiring surgical stabilization (Kim et al., 2018; Qadir et al., 2020; Shang et al., 2025). The perioperative period in these patients carries a high risk of venous thromboembolism (VTE), with deep vein thrombosis (DVT) being a common and serious complication (Samuel et al., 2018; Lv et al., 2024a; Wang et al., 2025). Despite standardized pharmacologic prophylaxis, the postoperative DVT rate following high-energy spinal fractures approaches 20%, a figure significantly higher than in many general orthopedic trauma populations (Lv et al., 2024b). This elevated risk is evident even preoperatively, with a reported DVT prevalence of approximately 14.5% in patients with thoracolumbar fractures (Wang et al., 2020; Ma et al., 2024). Consequences range from fatal pulmonary embolism to the long-term sequelae of post-thrombotic syndrome (Kahn & Ginsberg, 2002; Khan et al., 2021; Linnemann et al., 2024). Accurate preoperative identification of high-risk individuals is crucial for implementing targeted prophylactic strategies.

Current clinical practice often relies on generic VTE risk assessment tools, such as the Caprini score (Caprini et al., 1991). Although useful for broad surgical populations, these tools lack optimal precision in high-risk subgroups like patients with traumatic spinal fractures, as they fail to systematically incorporate key disease-specific determinants (Bilgi et al., 2016; Ratnasekera et al., 2023; Katiyar et al., 2023). Notably, the American Spinal Injury Association (ASIA) Impairment Scale—a gold-standard measure of neurological deficit that correlates directly with the degree of venous stasis and may reflect neurogenic influences on coagulation—is not integrated into these models (Tran et al., 2024). Furthermore, readily accessible preoperative biomarkers with well-established prognostic value for hypercoagulability (*e.g.*, D-dimer and fibrinogen) are underutilized in existing prediction paradigms (Geerts et al., 2008).

This study aimed to develop and internally validate a novel preoperative prediction model that integrates key demographic, injury-specific (including ASIA grade), and biomarker data to stratify postoperative lower extremity DVT. We constructed an accessible nomogram to guide personalized prophylactic decision-making, with the ultimate goal of improving outcomes in this vulnerable population.

## Materials and methods

### Study design and population

This retrospective cohort study was conducted at The People’s Hospital of Lichuan City. The hospital’s Medical Ethics Committee approved the study protocol (Approval No.: 2025003). The requirement for informed consent was waived due to the retrospective nature of the study and the use of anonymized data, in compliance with the Declaration of Helsinki. We screened the electronic health records of all consecutive patients admitted between January 2020 and March 2025 who underwent surgical intervention for acute traumatic cervical, thoracic, or lumbar spine fractures.

The inclusion criteria for this study were as follows: (1) age ≥18 years; (2) radiologically confirmed acute traumatic spinal fracture; (3) receipt of open reduction and internal fixation surgery; (4) completion of a comprehensive postoperative lower extremity venous duplex ultrasonography screening protocol within the first postoperative week; and (5) medical records with ≥80% completeness for a predefined set of 29 candidate predictor variables (*i.e.,* non-missing data for ≥24 of 29 variables). Patients with missing data on the primary outcome (DVT status) were excluded. The exclusion criteria for this study were as follows: (1) preoperative diagnosis of DVT or pulmonary embolism; (2) pre-existing severe coagulopathy or ongoing long-term therapeutic anticoagulation; (3) active malignancy or documented systemic autoimmune diseases; (4) concomitant pelvic or lower extremity fractures requiring surgical intervention; (5) incomplete essential data or loss to follow-up prior to outcome assessment; and (6) missing data for any of the final model predictors (excluded *via* listwise deletion; no imputation was performed).

### DVT surveillance and prophylaxis protocol

The primary outcome, postoperative lower extremity DVT, was diagnosed using compression duplex ultrasonography. All ultrasound examinations were performed and interpreted by board-certified radiologists blinded to the patients’ detailed clinical risk profiles. In equivocal cases, computed tomography venography (CTV) was used for confirmation. The protocol included a preoperative baseline ultrasound. Postoperatively, all patients underwent scheduled ultrasonography on days 1, 3, and 7 postoperatively, plus daily clinical assessments. Interobserver variability was minimized using standardized diagnostic criteria; in cases of discrepancy, a final review was conducted by a senior radiologist. DVT was categorized as proximal (involving the popliteal vein or above) or distal (involving the calf veins).

Perioperative thromboprophylaxis followed a standardized institutional protocol based on the Caprini Risk Assessment Model (RAM). Low-risk patients (Caprini score ≤3) received mechanical prophylaxis with intermittent pneumatic compression (IPC) devices. Moderate-to-high-risk patients (Caprini score ≥4) received combined mechanical prophylaxis and pharmacological anticoagulation, predominantly with low-molecular-weight heparin (LMWH; enoxaparin 4,000 IU subcutaneously once daily, with dose adjustment for renal impairment). Pharmacological prophylaxis was initiated 6–12 h postoperatively and continued for a minimum of 14 days, or until 48 h after the patient achieved significant independent ambulation. Adherence was monitored *via* nursing medication administration records.

### Data collection and candidate predictors

Data were extracted by trained research staff using a standardized electronic case report form (eCRF). Variables were predefined based on clinical relevance and literature review, covering four domains: (1) Demographics and Baseline Characteristics: Age, sex, body mass index (BMI), smoking status (current *vs.* non-smoker), alcohol consumption (regular drinker (≥210 g/week) *vs.* non-regular drinker); (2) Comorbidities: Coronary artery disease, hypertension, diabetes mellitus, cerebrovascular disease, chronic obstructive pulmonary disease (COPD)/pulmonary fibrosis, lower extremity vascular disease (documented history of peripheral arterial disease, chronic venous insufficiency, or prior superficial or deep vein thrombosis); (3) Injury & Perioperative Factors: Fracture location (cervical, thoracic, lumbar), injury mechanism (high-energy: *e.g.*, fall from height, motor vehicle collisions; low-energy: *e.g.*, simple falls), neurological status (American Spinal Injury Association (ASIA) Impairment Scale Grades A–E), surgical approach (internal fixation *vs.* decompression and fixation), total operative time (minutes), estimated intraoperative blood loss (mL), receipt of perioperative allogeneic blood transfusion (yes/no), preoperative bed rest duration (hours from admission to surgery: ≤72 *vs.* >72 h); (4) Preoperative Laboratory Parameters (measured within 24 h before surgery): D-dimer (mg/L), fibrinogen (FIB, g/L), prothrombin time (PT, s), activated partial thromboplastin time (APTT, s), platelet count (PLT, 10^9^/L), serum albumin (ALB, g/L), hemoglobin (Hb, g/L), C-reactive protein (CRP, mg/L), white blood cell count (WBC, 10^9^/L).

### Statistical analysis

Statistical analyses were performed using SPSS (v26.0), R (v4.3.1; R Core Team, 2023), and Zstats software (http://www.zstats.net). Continuous variables were described as mean ± standard deviation or median (interquartile range, IQR) based on distribution (Shapiro–Wilk test), and compared using Student’s *t*-test or the Mann–Whitney U test. Categorical variables were reported as frequencies (percentages) and compared using the Chi-square test or Fisher’s exact test.

The cohort was randomly split into a training set (70%, *n* = 1,175) for model development and a testing set (30%, *n* = 501) for model validation. With 239 DVT events and six predictors in the final model, the events per variable (EPV) was 39.8, which is well above the heuristic threshold of 10, indicating a low risk of model overfitting. This was further mitigated by robust internal validation using bootstrap resampling. In the training set, univariate logistic regression was used to identify candidate predictors with *P* < 0.1. Multicollinearity was assessed using the Generalized Variance Inflation Factor (GVIF); all values were <2.5, indicating no significant collinearity. A multivariable logistic regression model was constructed using backward stepwise selection based on the Akaike Information Criterion (AIC) to obtain a parsimonious final model. Continuous predictors (age, D-dimer, fibrinogen) were initially modeled as linear terms after confirming linearity in the log-odds *via* Martingale residual plots. Sensitivity analyses using restricted cubic splines did not improve model fit (likelihood ratio test, *P* > 0.10), supporting linear form for clinical utility. Variables with *P* < 0.05 in the final model were retained as independent predictors. Adjusted odds ratios (aOR) with 95% confidence intervals (CI) were reported.

The final model was visualized as a nomogram. Discriminatory ability was evaluated using AUC. Calibration was assessed *via* calibration plots and the Hosmer-Lemeshow goodness-of-fit test. Clinical utility was evaluated using decision curve analysis (DCA). Bootstrap resampling (1,000 repetitions) on the entire dataset provided bias-corrected performance estimates to address potential overfitting.

## Results

### Baseline demographics and characteristics

The final study cohort included 1,676 patients, of whom 239 (14.3%) developed postoperative lower extremity DVT. The baseline characteristics of the entire cohort, stratified by DVT status, are presented in Table 1. Patients with DVT were significantly older, had higher rates of pre-existing lower extremity vascular disease and severe neurological deficits (ASIA Impairment Scale grades A/B), and were more likely to have prolonged preoperative bed rest (>72 h). Preoperative D-dimer and fibrinogen levels were also markedly elevated in the DVT group (all *P* < 0.001). Among DVT cases, 167 (69.9%) were distal and 72 (30.1%) were proximal. No significant differences were observed between groups in gender, body mass index (BMI) smoking status, alcohol consumption, other comorbidities, fracture location, injury mechanism, surgical approach, transfusion requirement, or other laboratory parameters (all *P* > 0.05). The training and testing sets were well balanced across all baseline variables (all *P* > 0.05; Table S1). The baseline characteristics of the training set, stratified by DVT status, are presented in Table S2, reflecting the same significant predictors as the overall sets.

**Table 1:** Baseline characteristics.

**Variables**	**Total (*n* = 1,676)**	**Non-DVT (*n* = 1,437)**	**DVT (*n* = 239)**	**Statistic**	***P***
Gender, n (%)				*χ*^2^= 0.283	0.595
Female	696 (41.53)	593 (41.27)	103 (43.10)		
Male	980 (58.47)	844 (58.73)	136 (56.90)		
Age, M (Q_1_, Q_3_)	52.00 (42.00, 63.00)	51.00 (41.00, 62.00)	60.00 (49.00, 67.50)	*Z* = − 6.532	<0.001
BMI (Kg/m^2^), n (%)				*χ*^2^= 4.426	0.219
≤18.4	113 (6.74)	91 (6.33)	22 (9.21)		
18.5–23.9	843 (50.30)	721 (50.17)	122 (51.05)		
24.0–27.9	518 (30.91)	445 (30.97)	73 (30.54)		
≥28.0	202 (12.05)	180 (12.53)	22 (9.21)		
Smoking status, n (%)				*χ*^2^= 0.606	0.436
Non-smoker	1,295 (77.27)	1,115 (77.59)	180 (75.31)		
current	381 (22.73)	322 (22.41)	59 (24.69)		
Alcohol consumption, n (%)				*χ*^2^= 0.080	0.777
Non-regular drinker	1,316 (78.52)	1,130 (78.64)	186 (77.82)		
Regular drinker	360 (21.48)	307 (21.36)	53 (22.18)		
Coronary artery disease, n (%)				*χ*^2^= 1.191	0.275
No	1,541 (91.95)	1,317 (91.65)	224 (93.72)		
Yes	135 (8.05)	120 (8.35)	15 (6.28)		
Hypertension, n (%)				*χ*^2^= 0.197	0.657
No	1,193 (71.18)	1,020 (70.98)	173 (72.38)		
Yes	483 (28.82)	417 (29.02)	66 (27.62)		
Diabetes, n (%)				*χ*^2^= 0.002	0.967
No	1,446 (86.28)	1,240 (86.29)	206 (86.19)		
Yes	230 (13.72)	197 (13.71)	33 (13.81)		
Cerebrovascular disease, n (%)				*χ*^2^= 0.281	0.596
No	1,583 (94.45)	1 359 (94.57)	224 (93.72)		
Yes	93 (5.55)	78 (5.43)	15 (6.28)		
COPD or pulmonary fibrosis, n (%)				*χ*^2^= 1.368	0.242
No	1,608 (95.94)	1,382 (96.17)	226 (94.56)		
Yes	68 (4.06)	55 (3.83)	13 (5.44)		
Lower extremity vascular disease, n (%)				*χ*^2^= 59.155	<0.001
No	1,486 (88.66)	1,309 (91.09)	177 (74.06)		
Yes	190 (11.34)	128 (8.91)	62 (25.94)		
Fracture location, n (%)				*χ*^2^= 1.924	0.382
Cervical	336 (20.05)	284 (19.76)	52 (21.76)		
Thoracic	499 (29.77)	422 (29.37)	77 (32.22)		
Lumbar	841 (50.18)	731 (50.87)	110 (46.03)		
Injury mechanism, n (%)				*χ*^2^= 3.417	0.065
Low-energy injury	646 (38.54)	541 (37.65)	105 (43.93)		
High-energy injury	1,030 (61.46)	896 (62.35)	134 (56.07)		
ASIA grade, n (%)				*χ*^2^= 228.419	<0.001
A	137 (8.17)	69 (4.80)	68 (28.45)		
B	134 (8.00)	90 (6.26)	44 (18.41)		
C	178 (10.62)	143 (9.95)	35 (14.64)		
D	466 (27.80)	434 (30.20)	32 (13.39)		
E	761 (45.41)	701 (48.78)	60 (25.10)		
Surgical approach, n (%)				*χ*^2^= 0.205	0.650
Internal Fixation	1,164 (69.45)	1,001 (69.66)	163 (68.20)		
Decompression + Internal Fixation	512 (30.55)	436 (30.34)	76 (31.80)		
Blood transfusion, n (%)				*χ*^2^= 0.124	0.724
No	1,305 (77.86)	1,121 (78.01)	184 (76.99)		
Yes	371 (22.14)	316 (21.99)	55 (23.01)		
Total Operative Time (min) , M (Q_1_, Q_3_)	134.00 (110.00, 166.00)	133.00 (109.00, 164.00)	144.00 (116.50, 178.00)	*Z* = − 3.066	0.002
Intraoperative blood loss (ml), M (Q_1_, Q_3_)	230.00 (147.50, 300.00)	230.00 (140.00, 290.00)	250.00 (160.00, 320.00)	*Z* = − 1.979	0.048
Preoperative bed rest time, n (%)				*χ*^2^= 131.854	<0.001
≤72 h	1,135 (67.72)	1,050 (73.07)	85 (35.56)		
>72 h	541 (32.28)	387 (26.93)	154 (64.44)		
D-Dimer (mg/L), M (Q_1_, Q_3_)	1.80 (1.00, 3.40)	1.60 (1.00, 2.80)	5.30 (2.95, 7.65)	*Z* = − 16.366	<0.001
FIB (g/L), M (Q_1_, Q_3_)	4.20 (3.60, 4.90)	4.20 (3.50, 4.80)	4.70 (4.00, 5.30)	*Z* = − 6.374	<0.001
PT (s), M (Q_1_, Q_3_)	12.30 (11.10, 13.40)	12.30 (11.10, 13.40)	12.30 (11.20, 13.35)	*Z* = − 0.471	0.638
APTT (s), M (Q_1_, Q_3_)	29.65 (23.80, 35.90)	29.80 (24.00, 35.80)	29.00 (22.90, 36.75)	*Z* = − 0.515	0.606
PLT (10^9^ /L), M (Q_1_, Q_3_)	303.93 (226.72, 378.36)	303.89 (227.05, 378.27)	306.93 (225.69, 377.13)	*Z* = − 0.196	0.844
ALB (g/L), M (Q_1_, Q_3_)	34.87 (31.43, 38.53)	34.87 (31.40, 38.69)	34.80 (31.51, 37.67)	*Z* = − 1.202	0.229
Hb (g/L), M (Q_1_, Q_3_)	108.30 (97.10, 120.53)	108.80 (97.40, 120.70)	105.70 (95.15, 119.65)	*Z* = − 1.473	0.141
CRP (mg/L), M (Q_1_, Q_3_)	26.35 (15.41, 37.31)	26.19 (15.50, 37.26)	27.39 (13.50, 37.50)	*Z* = − 0.238	0.812
WBC (10^9^ /L), M (Q_1_, Q_3_)	11.00 (9.10, 13.00)	11.00 (9.10, 12.90)	11.20 (9.20, 13.10)	*Z* = − 0.833	0.405

**Notes.**

Z Mann–Whitney U test *χ*^2^ Chi-square test M Median Q_1_ 1st Quartile Q_3_ 3rd Quartile BMI body mass index COPD chronic obstructive pulmonary disease ASIA grade American Spinal Injury Association grade FIB fibrinogen PT prothrombin time APTT activated partial thromboplastin time PLT platelet count ALB serum albumin Hb hemoglobin CRP C-reactive protein WBC white blood cell count

### Identification of independent predictors

In the training set, univariate logistic regression identified several candidate predictors with *P* < 0.10 (Table 2). Backward stepwise selection based on AIC yielded a final model with six independent predictors (Table 3). The strongest predictor was preoperative bed rest exceeding 72 h (aOR = 5.208, 95% CI [3.319–8.171]; *P* < 0.001). Other significant predictors included pre-existing lower extremity vascular disease (aOR = 2.938, 95% CI [1.641–5.258]; *P* < 0.001), elevated preoperative D-dimer levels (aOR = 1.582 per mg/L, 95% CI [1.448–1.729]; *P* < 0.001), elevated preoperative fibrinogen levels (aOR = 1.434 per g/L, 95% CI [1.138–1.807]; *P* = 0.002), severe neurological impairment (ASIA grade A/B; with an inverse graded correlation with higher ASIA grades), and advanced age (aOR = 1.019 per year, 95% CI [1.003–1.035]; *P* = 0.018).

**Table 2:** Univariate analysis of risk factors for lower extremity DVT following traumatic spinal fracture surgery.

**Variables**	***P***	**OR (95% CI)**
Age, M (Q_1_, Q_3_)	<0.001	1.025 (1.013 ∼ 1.037)
BMI (Kg/m^2^), n (%)		
≤18.4		1.000 (Reference)
18.5–23.9	0.431	0.778 (0.417 ∼ 1.452)
24.0–27.9	0.170	0.631 (0.327 ∼ 1.219)
≥28.0	0.088	0.502 (0.228 ∼ 1.107)
Lower extremity vascular disease, n (%)		
No		1.000 (Reference)
Yes	<0.001	3.579 (2.358 ∼ 5.433)
ASIA grade, n (%)		
A		1.000 (Reference)
B	0.024	0.504 (0.278 ∼ 0.912)
C	<0.001	0.225 (0.124 ∼ 0.411)
D	<0.001	0.068 (0.038 ∼ 0.124)
E	<0.001	0.082 (0.049 ∼ 0.136)
Preoperative bed rest time, n (%)		
≤72 h		1.000 (Reference)
>72 h	<0.001	5.391 (3.798 ∼ 7.651)
D-Dimer (mg/L), M (Q_1_, Q_3_)	<0.001	1.637 (1.516 ∼ 1.767)
FIB (g/L), M (Q_1_, Q_3_)	<0.001	1.622 (1.360 ∼ 1.935)

**Notes.**

OR odds ratio CI Confidence Interval BMI body mass index ASIA grade American Spinal Injury Association grade FIB fibrinogen

**Table 3:** Multivariate logistic regression analysis of risk factors for lower extremity DVT following traumatic spinal fracture surgery.

**Variables**	*β*	**S.E**	**Z**	***P***	**OR (95% CI)**
Intercept	−5.033	0.747	−6.738	<0.001	0.007 (0.002 ∼ 0.028)
D-Dimer (mg/L)	0.459	0.045	10.155	<0.001	1.582 (1.448 ∼ 1.729)
Preoperative bed rest time					
≤ 72 h					1.000 (Reference)
>72 h	1.650	0.230	7.181	<0.001	5.208 (3.319 ∼ 8.171)
ASIA grade					
A					1.000 (Reference)
B	−1.104	0.410	−2.692	0.007	0.332 (0.148 ∼ 0.741)
C	−1.454	0.388	−3.753	<0.001	0.234 (0.109 ∼ 0.499)
D	−2.661	0.380	−7.009	<0.001	0.070 (0.033 ∼ 0.147)
E	−2.338	0.330	−7.088	<0.001	0.097 (0.051 ∼ 0.184)
Lower extremity vascular disease					
No					1.000 (Reference)
Yes	1.078	0.297	3.628	<0.001	2.938 (1.641 ∼ 5.258)
FIB (g/L)	0.360	0.118	3.053	0.002	1.434 (1.138 ∼ 1.807)
Age	0.019	0.008	2.372	0.018	1.019 (1.003 ∼ 1.035)

**Notes.**

OR odds ratio CI Confidence Interval ASIA grade American Spinal Injury Association grade FIB fibrinogen

### Nomogram construction and validation

**Figure 1:**
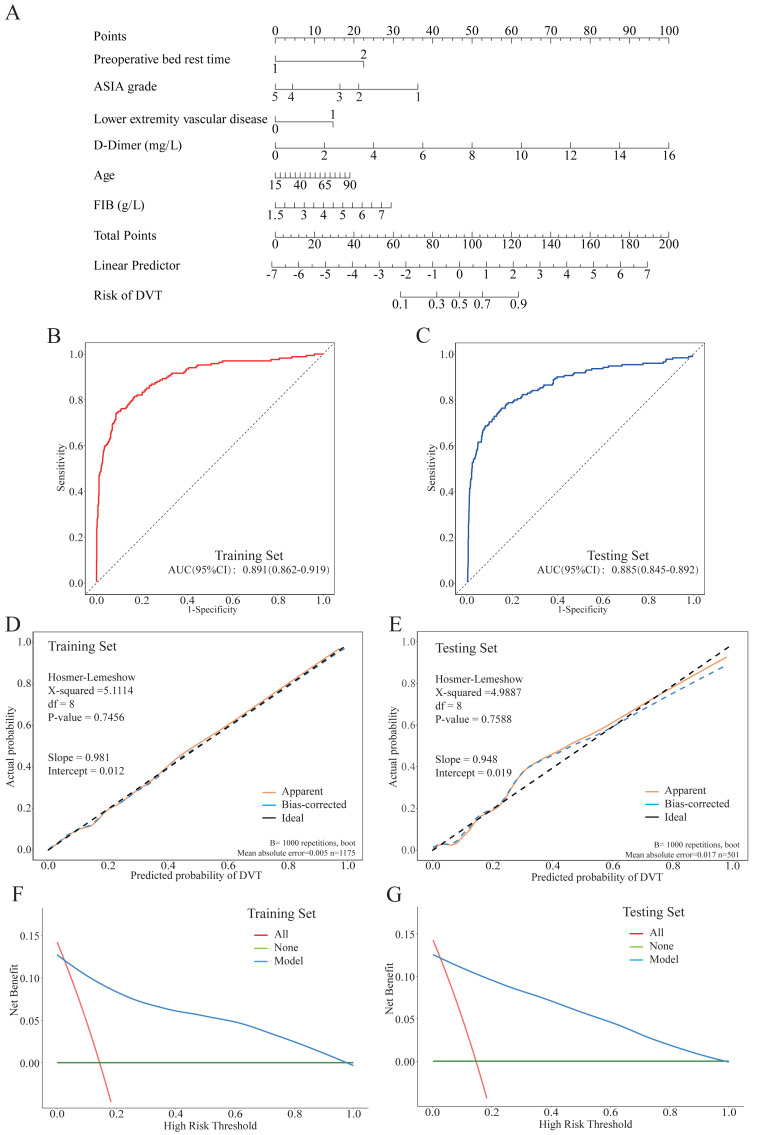
Development and validation of a nomogram for predicting postoperative lower extremity deep vein thrombosis (DVT) after traumatic spinal fracture surgery. (A) The nomogram integrates six preoperative predictors. The total points correspond to an individual’s predicted probability of DVT. (B, C) Receiver operating characteristic (ROC) curves of the model in the (B) training and (C) testing sets. (D, E) Calibration plots for the (D) training and (E) testing sets, comparing predicted probabilities against actual probabilities. The dashed black line represents perfect calibration. The orange line (“Apparent”) and blue line (“Bias-corrected”, 1,000 bootstrap repetitions) show the model’s calibration. (F, G) DCA for the (F) training and (G) testing sets. The net benefit of using the nomogram (blue line) is plotted against the strategies of treating all patients (red line) and treating no patients (green line) across a range of clinical threshold probabilities.

A preoperative nomogram was constructed based on the final multivariable logistic regression model to visually estimate individual postoperative DVT risk (Fig. 1A). The nomogram showed robust, consistent discriminative performance: AUC = 0.891 (95% CI [0.862–0.919]) in the training set and 0.885 (95% CI [0.849–0.921]) in the testing set (Figs. 1B, 1C). Bootstrap internal validation yielded an optimism-corrected AUC of 0.882, indicating minimal overfitting. Calibration was excellent, with slopes and intercepts close to ideal values; the Hosmer-Lemeshow test confirmed good fit (training: *χ*^2^ = 5.111, *df* = 8, *P* = 0.746; testing: *χ*^2^ = 4.989, *df* = 8, *P* = 0.759) (Figs. 1D, 1E). The model maintained high sensitivity (91.2% training, 90.5% testing) and moderate specificity (74.3% in the training set, 74.5% in the testing set) (Table S3). DCA confirmed the nomogram’s clinical utility across a wide range of threshold probabilities (approximately 2% to 97%), with higher net benefit than “treat-all” or “treat-none” strategies (Figs. 1F, 1G).

## Discussion

This study successfully developed and internally validated a preoperative nomogram for predicting postoperative lower extremity DVT in patients undergoing surgery for traumatic spinal fractures. The model incorporating six readily available preoperative variables—prolonged bed rest > 72 h, pre-existing lower extremity vascular disease, elevated D-dimer, elevated fibrinogen, severe neurological impairment (ASIA grade A/B), and advanced age—demonstrated robust discriminatory performance (area under the curve (AUC) = 0.885) and good calibration. DCA verified its clinical utility across clinically relevant risk thresholds. This tool addresses a critical need for specialized risk stratification in a population for whom generic assessment models fall short.

The need for a dedicated prediction model in traumatic spinal fracture patients is underscored by the limitations of applying generic VTE risk assessment tools to this population. The Caprini Risk Assessment Model (RAM), while widely used, shows inadequate discrimination in this population due to its failure to capture disease-specific risk determinants. A comparative study focusing on this specific population demonstrated that a logistic regression model based solely on the Caprini RAM exhibited poor performance (AUC = 0.595) in predicting postoperative DVT progression (Lai et al., 2024). This aligns with our rationale. The Caprini RAM, while comprehensive, lacks specificity for key disease-specific risk determinants, such as precise quantification of neurological deficit. Our model directly integrates the ASIA Impairment Scale, a cornerstone for assessing spinal cord injury severity. Identifying ASIA grades A/B as major independent risk factor (aOR = 5.208) quantifies and reinforces the well-established clinical association between severe neurological injury and VTE risk. This finding is consistent with prior research on spinal fracture patients, which also identified ASIA grades A and B as significant independent predictors for DVT (Wang et al., 2022a). The underlying pathophysiology extends beyond simple immobility, involving a complex neurogenic prothrombotic state mediated by sympathetic dysfunction, endothelial activation, and altered fibrinolytic activity—mechanisms not captured by generic models (Miranda & Hassouna, 2000). Our model also integrates D-dimer and fibrinogen, providing a direct biochemical snapshot of trauma-induced hypercoagulability and hyperfibrinolysis. While other spinal surgery prediction models exist (Yan et al., 2022; Lai et al., 2024), none combine these key coagulation biomarkers with the critical ASIA Impairment Scale, likely explaining our model’s superior discriminatory ability. This integrated approach may explain its seemingly superior discriminatory ability (AUC 0.885) compared to other reported models for high-energy spinal fractures (Wang et al., 2020; Wang et al., 2022b). Notably, studies on thoracolumbar fractures have shown that D-dimer levels exhibit a dynamic postoperative course, with levels on postoperative day 3 holding the highest diagnostic value for DVT, and that D-dimer elevation is significantly influenced by both operative time and ASIA Impairment Scale score (Wang et al., 2021; Wang et al., 2022a)—factors that our model accounts for either directly or indirectly through correlated variables.

The nomogram’s predictors are deeply rooted in Virchow’s triad, as this triad manifests in traumatic spinal injury, and support a proactive clinical strategy. Preoperative bed rest > 72 hours—the strongest predictor—directly reflects venous stasis. This finding emphasizes the preoperative period’s importance, highlighting that thrombotic risk increases with each additional day of preoperative immobilization. It supports expediting surgical planning and initiating aggressive mechanical prophylaxis immediately upon admission, consistent with data linking delayed intervention to higher DVT incidence (Sanchez et al., 2021; Lv et al., 2023). The nomogram translates this risk factor into an actionable metric, allowing clinicians to identify patients for whom minimizing every hour of preoperative stasis is paramount.

Elevated D-dimer and fibrinogen reflect trauma-induced hypercoagulability and hyperfibrinolysis (Xu et al., 2020; Nakae et al., 2022). Fibrinogen elevation indicates a prothrombotic acute-phase response, while D-dimer elevation reflects concurrent fibrinolysis and thrombus turnover (Mlačo et al., 2023; Collins et al., 2025). Their combined elevation enhances risk assessment precision. Notably, D-dimer dynamics in this population are strongly influenced by neurological injury severity and operative time (An, Huang & Wang, 2020; Wang et al., 2022a), making it an integrative biomarker capturing the injury’s inherent prothrombotic drive and anticipated surgical stress. This synergizes with the inclusion of severe neurological deficit (ASIA grades A/B), which extends beyond immobility. Severe spinal cord injury induces a distinct neurogenic prothrombotic state involving sympathetic dysfunction and endothelial activation—a key biological differentiator not captured by generic models and corroborated as a significant independent predictor in prior studies (Miranda & Hassouna, 2000; Mackiewicz-Milewska et al., 2021; Park et al., 2024).

Pre-existing lower extremity vascular disease and advanced age reflect baseline susceptibility, compounding acute injury-induced risk by predisposing patients to endothelial dysfunction and reduced vascular compliance (Nilsson, 2014; Grunewald et al., 2021; Boelitz et al., 2024). The model’s strength lies in synthesizing these preoperative variables (spanning stasis, hypercoagulability, and endothelial factors) to enable prospective risk stratification at initial assessment. This shifts practice from uniform prophylaxis to personalized, risk-adapted management. For high-risk patients, clinicians can consider early pharmacological intervention (when bleeding risk permits), intensify mechanical prophylaxis and monitoring, and counsel patients—aligning perioperative care with precision medicine principles.

This study has limitations. The single-center, retrospective design may introduce selection bias and limit generalizability. External validation across diverse populations and settings is essential to confirm transportability. While duplex ultrasonography is the clinical standard, its variable sensitivity for asymptomatic distal thrombi may impact outcome ascertainment. We used stepwise regression for variable selection. While this yielded a parsimonious, clinically applicable model, stepwise regression has known limitations regarding stability and overfitting risk. Robust bootstrap validation mitigates these concerns. Future studies should explore penalized regression (*e.g.*, least absolute shrinkage and selection operator (LASSO)) using larger multicenter datasets to optimize variable selection and enhance generalizability. The nomogram is a preoperative tool guiding decision-making at initial admission. While this facilitates early triage, it uses only preoperative variables and excludes dynamic intra- or postoperative factors, limiting its utility for continuous perioperative risk assessment. Prospective studies should assess whether integrating the nomogram into care pathways improves outcomes and optimizes resource use. Finally, methodological evolution, including the exploration of advanced modeling techniques and novel biomarkers, could further enhance predictive precision beyond this foundational, clinically oriented tool.

## Conclusion

This study developed and validated a clinically applicable preoperative nomogram for stratifying postoperative DVT risk in patients with traumatic spinal fractures. By integrating injury-specific, biomarker-related, and clinical factors, the model serves as a tailored alternative to generic risk assessment tools. It provides a framework for personalized preoperative decision-making, potentially improving outcomes through early identification of high-risk patients.

##  Supplemental Information

10.7717/peerj.21184/supp-1 Supplemental Information 1 Raw data

10.7717/peerj.21184/supp-2 Supplemental Information 2 Assessment of Equilibrium Between Training and Testing sets

10.7717/peerj.21184/supp-3 Supplemental Information 3 Baseline Characteristics of the Training Set

10.7717/peerj.21184/supp-4 Supplemental Information 4 Confusion Matrix for Training and Testing Sets

## Abbreviations

AIC: Akaike Information Criterion
ALB: Albumin
APTT: Activated Partial Thromboplastin Time
ASIA: American Spinal Injury Association
AUC: Area Under the Curve
BMI: Body Mass Index
COPD: Chronic Obstructive Pulmonary Disease
CRP: C-Reactive Protein
DCA: Decision Curve Analysis
DVT: Deep Vein Thrombosis
FIB: Fibrinogen
GVIF: Generalized Variance Inflation Factor
Hb: Hemoglobin
IQR: Interquartile Range
aOR: Adjusted odds ratio
PLT: Platelet Count
PT: Prothrombin Time
WBC: White Blood Cell Count

## Additional Information and Declarations

### Data Availability

The following information was supplied regarding data availability: The raw data is available in the Supplemental File.
