# Supplementary material for: Development and validation of a nomogram for predicting postoperative lower extremity deep vein thrombosis in patients with traumatic spinal fractures: a retrospective study
Source: PeerJ. 2026 Apr 22;14:e21184. doi: 10.7717/peerj.21184 (PMC13109978; doi:10.7717/peerj.21184)
Supplement: Supplemental Information 2 [file peerj-14-21184-s002.docx]

**Table S1** Assessment of Equilibrium Between Training and Testing sets

| **Variables** | **Total (n = 1676)** | **Testing (n = 501)** | **Training (n = 1175)** | **Statistic** | ***P*** |
| --- | --- | --- | --- | --- | --- |
| Gender, n(%) |  |  |  | χ²=0.013 | 0.909 |
| Female | 696 (41.53) | 207 (41.32) | 489 (41.62) |  |  |
| Male | 980 (58.47) | 294 (58.68) | 686 (58.38) |  |  |
| Age, M (Q₁, Q₃) | 52.00 (42.00, 63.00) | 52.00 (43.00, 62.00) | 52.00 (42.00, 64.00) | Z=-0.407 | 0.684 |
| BMI (Kg/m^2^), n(%) |  |  |  | χ²=1.277 | 0.735 |
| ≤18.4 | 113 (6.74) | 39 (7.78) | 74 (6.30) |  |  |
| 18.5–23.9 | 843 (50.30) | 251 (50.10) | 592 (50.38) |  |  |
| 24.0–27.9 | 518 (30.91) | 152 (30.34) | 366 (31.15) |  |  |
| ≥28.0 | 202 (12.05) | 59 (11.78) | 143 (12.17) |  |  |
| Smoking status, n(%) |  |  |  | χ²=0.562 | 0.453 |
| non-smoker | 1295 (77.27) | 393 (78.44) | 902 (76.77) |  |  |
| current | 381 (22.73) | 108 (21.56) | 273 (23.23) |  |  |
| Alcohol consumption, n(%) |  |  |  | χ²=0.325 | 0.569 |
| non-regular drinker | 1316 (78.52) | 389 (77.64) | 927 (78.89) |  |  |
| regular drinker | 360 (21.48) | 112 (22.36) | 248 (21.11) |  |  |
| Coronary artery disease, n(%) |  |  |  | χ²=0.729 | 0.393 |
| No | 1541 (91.95) | 465 (92.81) | 1076 (91.57) |  |  |
| Yes | 135 (8.05) | 36 (7.19) | 99 (8.43) |  |  |
| Hypertension, n(%) |  |  |  | χ²=0.402 | 0.526 |
| No | 1193 (71.18) | 362 (72.26) | 831 (70.72) |  |  |
| Yes | 483 (28.82) | 139 (27.74) | 344 (29.28) |  |  |
| Diabetes, n(%) |  |  |  | χ²=0.938 | 0.333 |
| No | 1446 (86.28) | 426 (85.03) | 1020 (86.81) |  |  |
| Yes | 230 (13.72) | 75 (14.97) | 155 (13.19) |  |  |
| Cerebrovascular disease, n(%) |  |  |  | χ²=0.556 | 0.456 |
| No | 1583 (94.45) | 470 (93.81) | 1113 (94.72) |  |  |
| Yes | 93 (5.55) | 31 (6.19) | 62 (5.28) |  |  |
| COPD or pulmonary fibrosis, n(%) |  |  |  | χ²=1.597 | 0.206 |
| No | 1608 (95.94) | 476 (95.01) | 1132 (96.34) |  |  |
| Yes | 68 (4.06) | 25 (4.99) | 43 (3.66) |  |  |
| Lower extremity vascular disease, n(%) |  |  |  | χ²=1.906 | 0.167 |
| No | 1486 (88.66) | 436 (87.03) | 1050 (89.36) |  |  |
| Yes | 190 (11.34) | 65 (12.97) | 125 (10.64) |  |  |
| Fracture location, n(%) |  |  |  | χ²=0.245 | 0.885 |
| Cervical | 336 (20.05) | 99 (19.76) | 237 (20.17) |  |  |
| Thoracic | 499 (29.77) | 146 (29.14) | 353 (30.04) |  |  |
| Lumbar | 841 (50.18) | 256 (51.10) | 585 (49.79) |  |  |
| Injury mechanism, n(%) |  |  |  | χ²=0.607 | 0.436 |
| Low-energy injury | 646 (38.54) | 186 (37.13) | 460 (39.15) |  |  |
| High-energy injury | 1030 (61.46) | 315 (62.87) | 715 (60.85) |  |  |
| ASIA grade, n(%) |  |  |  | χ²=0.864 | 0.930 |
| A | 137 (8.17) | 43 (8.58) | 94 (8.00) |  |  |
| B | 134 (8.00) | 44 (8.78) | 90 (7.66) |  |  |
| C | 178 (10.62) | 52 (10.38) | 126 (10.72) |  |  |
| D | 466 (27.80) | 136 (27.15) | 330 (28.09) |  |  |
| E | 761 (45.41) | 226 (45.11) | 535 (45.53) |  |  |
| Surgical approach, n(%) |  |  |  | χ²=1.639 | 0.201 |
| Internal Fixation | 1164 (69.45) | 359 (71.66) | 805 (68.51) |  |  |
| Decompression + Internal Fixation | 512 (30.55) | 142 (28.34) | 370 (31.49) |  |  |
| Blood transfusion, n(%) |  |  |  | χ²=0.073 | 0.787 |
| No | 1305 (77.86) | 388 (77.45) | 917 (78.04) |  |  |
| Yes | 371 (22.14) | 113 (22.55) | 258 (21.96) |  |  |
| Total Operative Time (min) , M (Q₁, Q₃) | 134.00 (110.00, 166.00) | 135.00 (109.00, 163.00) | 134.00 (110.00, 168.00) | Z=-0.648 | 0.517 |
| Intraoperative blood loss (ml), M (Q₁, Q₃) | 230.00 (147.50, 300.00) | 220.00 (150.00, 290.00) | 230.00 (140.00, 300.00) | Z=-0.962 | 0.336 |
| Preoperative bed rest time, n(%) |  |  |  | χ²=0.096 | 0.756 |
| ≤ 72 h | 1135 (67.72) | 342 (68.26) | 793 (67.49) |  |  |
| > 72 h | 541 (32.28) | 159 (31.74) | 382 (32.51) |  |  |
| D-Dimer(mg/L), M (Q₁, Q₃) | 1.80 (1.00, 3.40) | 1.90 (1.10, 3.40) | 1.80 (1.00, 3.40) | Z=-0.535 | 0.592 |
| FIB (g/L), M (Q₁, Q₃) | 4.20 (3.60, 4.90) | 4.30 (3.60, 5.00) | 4.20 (3.60, 4.90) | Z=-0.690 | 0.490 |
| PT (s), M (Q₁, Q₃) | 12.30 (11.10, 13.40) | 12.10 (10.90, 13.40) | 12.30 (11.10, 13.40) | Z=-1.635 | 0.102 |
| APTT (s), M (Q₁, Q₃) | 29.65 (23.80, 35.90) | 29.60 (23.90, 35.90) | 29.80 (23.80, 35.85) | Z=-0.143 | 0.886 |
| PLT (10^9^/L), M (Q₁, Q₃) | 303.93 (226.72, 378.36) | 311.01 (231.90, 376.41) | 300.91 (222.24, 378.90) | Z=-0.760 | 0.447 |
| ALB (g/L), M (Q₁, Q₃) | 34.87 (31.43, 38.53) | 34.75 (31.48, 38.42) | 34.90 (31.41, 38.54) | Z=-0.937 | 0.349 |
| Hb (g/L), M (Q₁, Q₃) | 108.30 (97.10, 120.53) | 108.70 (98.00, 121.50) | 108.20 (97.00, 120.05) | Z=-0.663 | 0.507 |
| CRP (mg/L), M (Q₁, Q₃) | 26.35 (15.41, 37.31) | 26.39 (15.26, 37.23) | 26.32 (15.48, 37.58) | Z=-0.093 | 0.926 |
| WBC (10^9^/L), M (Q₁, Q₃) | 11.00 (9.10, 13.00) | 10.90 (9.10, 12.80) | 11.10 (9.10, 13.00) | Z=-0.929 | 0.353 |
| Z: Mann-Whitney U test, χ²: Chi-square test, M: Median, Q₁: 1st Quartile, Q₃: 3rd Quartile, BMI: body mass index, COPD: chronic obstructive pulmonary disease, ASIA grade: American Spinal Injury Association grade, FIB: fibrinogen, PT: prothrombin time, APTT: activated partial thromboplastin time, PLT: platelet count, ALB: serum albumin, Hb: hemoglobin, CRP: C-reactive protein, WBC: white blood cell count. | | | | | |
