# Supplementary material for: Development and validation of a nomogram for predicting postoperative lower extremity deep vein thrombosis in patients with traumatic spinal fractures: a retrospective study
Source: PeerJ. 2026 Apr 22;14:e21184. doi: 10.7717/peerj.21184 (PMC13109978; doi:10.7717/peerj.21184)
Supplement: Supplemental Information 3 [file peerj-14-21184-s003.docx]

**Table S2** Baseline Characteristics of the Training Set

| **Variables** | **Total (n = 1175)** | **Non-DVT (n = 1008)** | **DVT (n = 167)** | **Statistic** | ***P*** |
| --- | --- | --- | --- | --- | --- |
| Gender, n(%) |  |  |  | χ²=0.352 | 0.553 |
| Female | 489 (41.62) | 423 (41.96) | 66 (39.52) |  |  |
| Male | 686 (58.38) | 585 (58.04) | 101 (60.48) |  |  |
| Age, M (Q₁, Q₃) | 52.00 (42.00, 64.00) | 51.00 (41.00, 63.00) | 60.00 (47.00, 68.00) | Z=-4.579 | **< 0.001** |
| BMI (Kg/m^2^), n(%) |  |  |  | χ²=4.187 | 0.242 |
| ≤18.4 | 74 (6.30) | 60 (5.95) | 14 (8.38) |  |  |
| 18.5–23.9 | 592 (50.38) | 501 (49.70) | 91 (54.49) |  |  |
| 24.0–27.9 | 366 (31.15) | 319 (31.65) | 47 (28.14) |  |  |
| ≥28.0 | 143 (12.17) | 128 (12.70) | 15 (8.98) |  |  |
| Smoking, n(%) |  |  |  | χ²=0.189 | 0.664 |
| Non-smoker | 902 (76.77) | 776 (76.98) | 126 (75.45) |  |  |
| current | 273 (23.23) | 232 (23.02) | 41 (24.55) |  |  |
| Alcohol consumption, n(%) |  |  |  | χ²=0.024 | 0.878 |
| non-regular drinker | 927 (78.89) | 796 (78.97) | 131 (78.44) |  |  |
| regular drinker | 248 (21.11) | 212 (21.03) | 36 (21.56) |  |  |
| Coronary artery disease, n(%) |  |  |  | χ²=0.104 | 0.747 |
| non-regular drinker | 1076 (91.57) | 922 (91.47) | 154 (92.22) |  |  |
| regular drinker | 99 (8.43) | 86 (8.53) | 13 (7.78) |  |  |
| Hypertension, n(%) |  |  |  | χ²=0.121 | 0.728 |
| No | 831 (70.72) | 711 (70.54) | 120 (71.86) |  |  |
| Yes | 344 (29.28) | 297 (29.46) | 47 (28.14) |  |  |
| Diabetes, n(%) |  |  |  | χ²=0.000 | 0.994 |
| No | 1020 (86.81) | 875 (86.81) | 145 (86.83) |  |  |
| Yes | 155 (13.19) | 133 (13.19) | 22 (13.17) |  |  |
| Cerebrovascular disease, n(%) |  |  |  | χ²=0.458 | 0.498 |
| No | 1113 (94.72) | 953 (94.54) | 160 (95.81) |  |  |
| Yes | 62 (5.28) | 55 (5.46) | 7 (4.19) |  |  |
| COPD or pulmonary fibrosis, n(%) |  |  |  | χ²=0.706 | 0.401 |
| No | 1132 (96.34) | 973 (96.53) | 159 (95.21) |  |  |
| Yes | 43 (3.66) | 35 (3.47) | 8 (4.79) |  |  |
| Lower extremity vascular disease, n(%) |  |  |  | χ²=39.636 | **< 0.001** |
| No | 1050 (89.36) | 924 (91.67) | 126 (75.45) |  |  |
| Yes | 125 (10.64) | 84 (8.33) | 41 (24.55) |  |  |
| Fracture location, n(%) |  |  |  | χ²=1.443 | 0.486 |
| Cervical | 237 (20.17) | 201 (19.94) | 36 (21.56) |  |  |
| Thoracic | 353 (30.04) | 298 (29.56) | 55 (32.93) |  |  |
| Lumbar | 585 (49.79) | 509 (50.50) | 76 (45.51) |  |  |
| Injury mechanism, n(%) |  |  |  | χ²=2.712 | 0.100 |
| Low-energy injury | 460 (39.15) | 385 (38.19) | 75 (44.91) |  |  |
| High-energy injury | 715 (60.85) | 623 (61.81) | 92 (55.09) |  |  |
| ASIA grade, n(%) |  |  |  | χ²=170.498 | **< 0.001** |
| A | 94 (8.00) | 46 (4.56) | 48 (28.74) |  |  |
| B | 90 (7.66) | 59 (5.85) | 31 (18.56) |  |  |
| C | 126 (10.72) | 102 (10.12) | 24 (14.37) |  |  |
| D | 330 (28.09) | 308 (30.56) | 22 (13.17) |  |  |
| E | 535 (45.53) | 493 (48.91) | 42 (25.15) |  |  |
| Surgical approach, n(%) |  |  |  | χ²=0.065 | 0.799 |
| Internal Fixation | 805 (68.51) | 692 (68.65) | 113 (67.66) |  |  |
| Decompression + Internal Fixation | 370 (31.49) | 316 (31.35) | 54 (32.34) |  |  |
| Blood transfusion, n(%) |  |  |  | χ²=0.221 | 0.638 |
| No | 917 (78.04) | 789 (78.27) | 128 (76.65) |  |  |
| Yes | 258 (21.96) | 219 (21.73) | 39 (23.35) |  |  |
| Total Operative Time (min) , M (Q₁, Q₃) | 134.00 (110.00, 168.00) | 133.00 (110.00, 166.00) | 144.00 (111.50, 179.50) | Z=-1.786 | 0.074 |
| Intraoperative blood loss (ml), M (Q₁, Q₃) | 230.00 (140.00, 300.00) | 230.00 (140.00, 300.00) | 230.00 (165.00, 340.00) | Z=-1.414 | 0.157 |
| Preoperative bed rest time, n(%) |  |  |  | χ²=102.300 | **< 0.001** |
| ≤ 72 h | 793 (67.49) | 737 (73.12) | 56 (33.53) |  |  |
| > 72 h | 382 (32.51) | 271 (26.88) | 111 (66.47) |  |  |
| D-Dimer (mg/L), M (Q₁, Q₃) | 1.80 (1.00, 3.40) | 1.60 (1.00, 2.80) | 5.20 (2.80, 7.65) | Z=-13.092 | **< 0.001** |
| FIB (g/L), M (Q₁, Q₃) | 4.20 (3.60, 4.90) | 4.20 (3.50, 4.80) | 4.70 (4.10, 5.30) | Z=-5.772 | **< 0.001** |
| PT (s), M (Q₁, Q₃) | 12.30 (11.10, 13.40) | 12.30 (11.10, 13.40) | 12.50 (11.30, 13.50) | Z=-1.328 | 0.184 |
| APTT (s), M (Q₁, Q₃) | 29.80 (23.80, 35.85) | 29.80 (23.98, 35.82) | 28.70 (23.00, 35.80) | Z=-0.259 | 0.795 |
| PLT (10^9^/L), M (Q₁, Q₃) | 300.91 (222.24, 378.90) | 300.70 (222.24, 378.12) | 301.06 (223.68, 382.60) | Z=-0.141 | 0.888 |
| ALB (g/L), M (Q₁, Q₃) | 34.90 (31.41, 38.54) | 34.95 (31.39, 38.74) | 34.74 (31.51, 37.50) | Z=-1.042 | 0.298 |
| Hb (g/L), M (Q₁, Q₃) | 108.20 (97.00, 120.05) | 108.40 (97.20, 120.23) | 106.10 (94.95, 119.85) | Z=-0.939 | 0.348 |
| CRP (mg/L), M (Q₁, Q₃) | 26.32 (15.48, 37.58) | 26.12 (15.46, 37.32) | 27.61 (16.50, 37.80) | Z=-0.352 | 0.725 |
| WBC (10^9^/L), M (Q₁, Q₃) | 11.10 (9.10, 13.00) | 11.00 (9.07, 13.00) | 11.50 (9.15, 13.10) | Z=-0.809 | 0.418 |
| Z: Mann-Whitney U test, χ²: Chi-square test, M: Median, Q₁: 1st Quartile, Q₃: 3rd Quartile, BMI: body mass index, COPD: chronic obstructive pulmonary disease, ASIA grade: American Spinal Injury Association grade, FIB: fibrinogen, PT: prothrombin time, APTT: activated partial thromboplastin time, PLT: platelet count, ALB: serum albumin, Hb: hemoglobin, CRP: C-reactive protein, WBC: white blood cell count. | | | | | |
