# Supplementary material for: Development and validation of a nomogram for predicting postoperative lower extremity deep vein thrombosis in patients with traumatic spinal fractures: a retrospective study
Source: PeerJ. 2026 Apr 22;14:e21184. doi: 10.7717/peerj.21184 (PMC13109978; doi:10.7717/peerj.21184)
Supplement: Supplemental Information 4 [file peerj-14-21184-s004.docx]

**Table S3** Confusion Matrix for Training and Testing Sets

| **Data** | **AUC (95%CI)** | **Accuracy (95%CI)** | **Sensitivity (95%CI)** | **Specificity (95%CI)** | **PPV (95%CI)** | **NPV (95%CI)** | **cut off** |
| --- | --- | --- | --- | --- | --- | --- | --- |
| Train | 0.891 (0.862-0.919) | 0.888 (0.868-0.905) | 0.912 (0.894-0.929) | 0.743 (0.676-0.809) | 0.955 (0.942-0.968) | 0.582 (0.516-0.648) | 0.241 |
| Test | 0.885 (0.849-0.921) | 0.870 (0.845-0.892) | 0.905 (0.884-0.926) | 0.745 (0.665-0.825) | 0.952 (0.934-0.970) | 0.625 (0.550-0.700) | 0.241 |
